# Trimester-Specific Bioelectrical Impedance Reference Curves in Physiological Pregnancy: Mapping Maternal Body Composition and Hydration Trajectories

**DOI:** 10.3390/nu18203341

**Published:** 2026-10-12

**Authors:** Daniela Denis Di Martino, Elisa Sabattini, Lucrezia Viscioni, Serena Cerri, Tatjana Radaelli, Sara Ornaghi, Jennifer Belfiore, Tamara Stampalija, Roberto Nuredini, Giulia Privitera, Gabriele Tinè, Enrico Ferrazzi, 3P Study Group Collaborators

**Affiliations:** 1 Department of Mother and Child, Mangiagalli Center, Fondazione IRCCS Ca’ Granda Ospedale Maggiore Policlinico, 20122 Milan, Italy; daniela.dimartino@asst-fbf-sacco.it (D.D.D.M.); serena.cerri96@gmail.com (S.C.); tatjana.radaelli@policlinico.mi.it (T.R.); giulia.privitera@policlinico.mi.it (G.P.); 2 Department of Clinical and Community Sciences, University of Milan, 20122 Milan, Italy; elisa.sabattini@unimi.it (E.S.); lucrezia.viscioni@unimi.it (L.V.); enrico.ferrazzi@unimi.it (E.F.); 3 Unit of Obstetrics, Fondazione IRCCS San Gerardo dei Tintori, 20900 Monza, Italy; sara.ornaghi@unimib.it; 4 School of Medicine and Surgery, University of Milano-Bicocca, 20900 Monza, Italy; 5 Department of Metabolism, Digestion and Reproduction, Institute of Reproductive and Developmental Biology, Imperial College London, London SW7 2AZ, UK; tamara.stampalija@gmail.com; 6 Department of Mother and Neonate, Institute for Maternal and Child Health IRCCS “Burlo Garofolo”, 34137 Trieste, Italy; roberto.nuredini@burlo.trieste.it; 7 Department of Economics, Quantitative Methods and Business Strategies, University of Milano-Bicocca, 20900 Milan, Italy; gabriele.tine@istitutotumori.mi.it

**Keywords:** bioelectrical impedance analysis, maternal body composition, pregnancy, gestational reference curves, total body water

## Abstract

**Background/Objectives:** Clinical risk measures such as pre-pregnancy body mass index (BMI) and gestational weight gain do not distinguish the expansion of fluid, lean, and adipose compartments. Standardized obstetric reference curves for bioelectrical impedance analysis (BIA) are lacking. This study aimed to establish longitudinal, trimester-specific BIA reference curves in uncomplicated pregnancies. **Methods:** This multicenter, prospective, longitudinal cohort study was conducted at three Italian tertiary research hospitals from September 2020 to September 2025. BIA assessments (InBody 270^®^) were performed at 10–15, 23–25, and 34–37 gestational weeks. Generalized additive models for location, scale, and shape were used to derive reference centiles and BMI-conditioned contour plots for total body water, lean mass, fat mass, and visceral fat level. Pregnancies with complications or incomplete longitudinal data were excluded from the primary reference cohort. **Results:** Among 442 healthy pregnant women, all parameters increased progressively across gestation. Each 1-unit increase in pre-pregnancy BMI was associated with a larger shift in fat mass (1.88 kg; 95% CI, 1.77–1.99) than in total body water (0.47 L; 95% CI, 0.38–0.57). **Conclusions:** These multicenter, BMI-conditioned, trimester-specific BIA reference curves provide normative benchmarks for maternal hydration and body composition and may support the identification of early deviations from physiological gestational trajectories.

## 1. Introduction

Pregnancy is characterized by major dynamic physiological adaptations encompassing metabolic shifts, cardiovascular remodeling, and a substantial expansion of total body water (TBW) [1]. While these modifications are pivotal to support fetal development, their deviance from a physiological trajectory might be intertwined with some gestational complications such as hypertensive disorders of pregnancy, fetal growth restriction and others [2,3,4,5]. Pre-pregnancy Body Mass Index (BMI) and Gestational Weight Gain (GWG) have been used as clinical risk factors to predict adverse outcomes, and to induce proactive interventions on maternal nutritional and lifestyle habits [6,7]. However, crude weight assessment exhibits a major limitation: it fails to differentiate between the expansion of distinct body compartments, namely fat mass (FM), fat-free mass (FFM), and fluid distribution [8].

Increasing clinical evidence highlights that maternal obesity and excessive GWG act as primary drivers for the establishment of a chronic, low-grade inflammatory state, frequently termed metabolic-inflammation, or meta-inflammation [8,9]. Hypertrophic adipose tissue undergoes cellular hypoxia and macrophage infiltration, triggering an accelerated systemic release of pro-inflammatory cytokines such as Tumor Necrosis Factor-alpha (TNF-alpha), Interleukin-6 (IL-6), and Interleukin-1 beta (IL-1 beta) [10,11,12]. This lipotoxic and inflammatory environment disrupts insulin signaling pathways, alters maternal endothelial function, and impairs placental nutrient transport [13,14,15,16]. Consequently, meta-inflammation significantly elevates the risk of developing gestational diabetes (GD), hypertensive disorders of pregnancy (HDP) including preeclampsia, and fetal growth aberrations ranging from restriction (FGR) to macrosomia [8,14,16].

To intercept these pathophysiological mechanisms before clinical symptoms manifest, a more sophisticated, compartment-specific assessment of maternal body composition is required. Bioelectrical Impedance Analysis (BIA) offers a non-invasive, safe, and highly reproducible method to estimate body fluid compartments and active tissue components [17,18,19].

While BIA holds significant prospective clinical potential to track fluid shifts and adipose tissue dynamics, its implementation in obstetrics has been limited by a lack of standardized, pregnancy-specific reference values. Standard BIA equations and reference vectors are traditionally calibrated on non-pregnant populations, failing to account for the unique, fluid-driven impedance shifts that characterize normal gestation [19,20,21,22,23]. Furthermore, existing pregnancy BIA literature has been constrained by single-center cohorts, small sample sizes, reliance on standard non-pregnant BIA vector equations, or a lack of rigorous post-hoc filtering of late-occurring pregnancy complications.

To bridge this gap, this study aims to establish longitudinal, trimester-specific bioelectrical impedance reference curves in uncomplicated pregnancies. By defining the normative trajectories of body composition and hydration status in a healthy cohort of pregnant women, these curves are intended to provide clinicians with a precise benchmark and robust reference values for bioelectric impedance parameters.

## 2. Materials and Methods

### 2.1. Study Design and Setting

This is a multicenter, prospective, observational study conducted across three Italian tertiary research hospitals: L. Mangiagalli Unit of Obstetrics, Fondazione IRCCS Ca’ Granda Ospedale Maggiore Policlinico (Milan, which served as the coordinating center); Unit of Obstetrics, Fondazione IRCCS San Gerardo dei Tintori (Monza); and Unit of Prenatal Diagnosis, IRCCS Burlo Garofalo Maternal and Child Health Hospital (Trieste). The study spanned a 5-year period, with recruitment and follow-up extending from September 2020 to September 2025.

The current study represents a dedicated sub-analysis of a larger, medical device observational master protocol entitled “*Early prediction of the different preeclampsia phenotypes based on demographic, biophysical, biochemical, impedance, and hemodynamic parameters*”. The study protocol was approved by the Institutional Review Board and Ethics Committee of the coordinating center (Mangiagalli, Milan) in September 2020 (Ethics Committee Approval No. 74_2020). All participants provided written informed consent prior to clinical enrollment.

### 2.2. Participants and Eligibility Criteria

Pregnant women were approached during their first-trimester combined screening, which integrates screening for fetal aneuploidies and preeclampsia.

To be eligible for enrollment into this study, patients had to meet the following inclusion criteria: age older than 18 years, singleton pregnancy, low-risk for fetal aneuploidies, absence of fetal malformations and provided signed informed consent. Patients with pre-existing chronic medical conditions that could alter the hemodynamic or metabolic profile (chronic hypertension, type I or type II diabetes mellitus, systemic autoimmune disorders, or documented inherited/acquired thrombophilia) were excluded.

### 2.3. Post Hoc Exclusion Criteria for the Normative Cohort

To establish normative reference curves strictly representative of uncomplicated gestations, a secondary post hoc filtering strategy was applied that excluded patients who developed any pregnancy-related complications after enrollment from this reference cohort. These complications included any of the following: HDP, FGR, GD or preterm birth (PTB). Patients who failed to complete the bioelectrical impedance assessments across all three main gestational time points were excluded due to incomplete longitudinal data.

### 2.4. Variables and Measurements

Eligible patients with uncomplicated pregnancies underwent three sequential clinical evaluations. Bioelectrical impedance and anthropometric assessments were performed longitudinally during three specific gestational age windows: first trimester (10–15 weeks of gestation), second trimester (23–25 weeks of gestation), and third trimester (34–37 weeks of gestation).

A final postpartum follow-up visit was systematically conducted to collect mode of delivery, neonatal birth weight and outcome, and final maternal-fetal data, thereby definitively confirming the physiological nature of the pregnancy before including the patient’s data in the normative reference pool. At each gestational visit, maternal weight and height were recorded.

Maternal body composition and raw bioelectrical parameters were assessed using a multi-frequency, multi-segmental body composition analyzer (InBody 270, InBody Co., Seoul, South Korea). The measurements were performed in accordance with the manufacturer’s clinical guidelines and standardized research protocols [24]. The assessment was executed with the patient in a stable, upright standing position on the scale platform; direct contact was established via an 8-point tactile electrode system embedded within the device, consisting of two foot-pad electrodes positioned under the soles of the feet and two handgrip electrodes held in the palms, with the thumbs placed on the upper sensors. Prior to electrode contact, the palms and soles were cleansed and moistened using specialized electrolyte tissues to minimize contact skin impedance. Patients were verified to have emptied their bladders before the assessment to avoid confounding fluid-volume artifacts. Direct electrical properties, including Resistance and Reactance, were captured across the device’s operational frequencies. The systemic Phase Angle was mathematically derived from the raw bioelectrical data.

It must be highlighted that BIA does not measure tissue mass or volume components directly. Parameters such as Total Body Water (TBW), Lean Mass (LM), Fat Mass (FM), and Visceral Fat Level (VF) represent device-derived estimates calculated using the manufacturer’s proprietary Direct Segmental Multi-frequency BIA (DSM-BIA) algorithms. Visceral Fat Level is expressed in arbitrary units (a.u., ranging on a scale from 1 to 20), serving as an index proportional to the estimated abdominal visceral fat cross-sectional area.

### 2.5. Statistical Analysis

Baseline patient characteristics and observed bioimpedance values were summarized as medians and interquartile ranges (IQRs) for continuous variables and as counts and percentages for categorical variables.

The primary objective was to derive gestational age-specific reference centile curves for maternal bioimpedance parameters during pregnancy. The primary endpoints were Total Body Water, Lean Mass, Fat Mass, and Visceral Fat level.

For each bioimpedance parameter, generalized additive models for location, scale, and shape (GAMLSS) were used to estimate gestational age-specific reference percentile curves. In the models, the dependent variable was the bioimpedance parameter of interest, and gestational age, pre-pregnancy BMI, and pre-pregnancy smoking status were evaluated as covariates of the location parameter. To allow smooth nonlinear trajectories of the bioimpedance parameters across pregnancy, gestational age was modeled using a restricted cubic spline with 3 internal knots, spanning the first, second, and third trimester. As the objective was to derive population reference centiles rather than predict participant-specific trajectories, the primary models characterized the overall distribution of bioimpedance values conditional on gestational age and maternal characteristics. Within-participant correlation was accounted for in the estimation of confidence intervals and *p*-values using participant-level bootstrap resampling. To assess the robustness of the estimated centiles, longitudinal sensitivity models were fitted using a Gaussian copula with an unstructured within-participant correlation matrix, retaining the same marginal distributions and covariate specifications as the primary models.

The conditional outcome distribution was selected by comparing converged candidate distributional models using the Bayesian information criterion (BIC). Candidate distributions included the normal, Box–Cox Cole-Green, Box–Cox power exponential, and Box–Cox t-distributions, allowing increasing flexibility for skewness and tail behavior. The scale parameter was evaluated as constant or gestational age-dependent, whereas shape parameters were kept constant.

For each endpoint, gestational age-specific reference centile curves were constructed using the fitted conditional distribution from the selected GAMLSS model. At each gestational age, the expected value was defined as the fitted 50th centile, and the lower and upper reference limits were defined as the fitted 5th and 95th centiles, respectively, corresponding to the central 90% reference interval. Additional intermediate centiles were estimated and used to display both conventional reference centile curves and contour plots of the fitted bioimpedance distribution across gestational age.

Model goodness of fit and calibration were assessed using normalized quantile residuals, observed-to-expected centile agreement, and empirical coverage of the central reference intervals. For each endpoint, the observed proportions below the 5th centile, between the 5th and 95th centiles, and above the 95th centile were compared with their nominal probabilities. Calibration was further evaluated across gestational age and pre-pregnancy BMI strata and recruiting centers.

A sensitivity analysis for between-center differences was performed by refitting the primary models with center included as an additional fixed-effect covariate in the location parameter. Fitted centile curves were compared with those from the primary analysis. To assess whether the reference trajectories were influenced by GD or other minor obstetric complications, an additional sensitivity analysis was performed including these pregnancies in the model-derivation population.

All analyses were performed using R version 4.4.1.

## 3. Results

### 3.1. Study Population

Out of the 938 patients initially enrolled at the first-trimester combined screening, 164 women diagnosed with major pregnancy complications during follow-up, including HDP, FGR and GD, were excluded. This led to a final study population of 774 pregnant women who were eligible as the baseline physiologic reference population.

**Figure 1:**
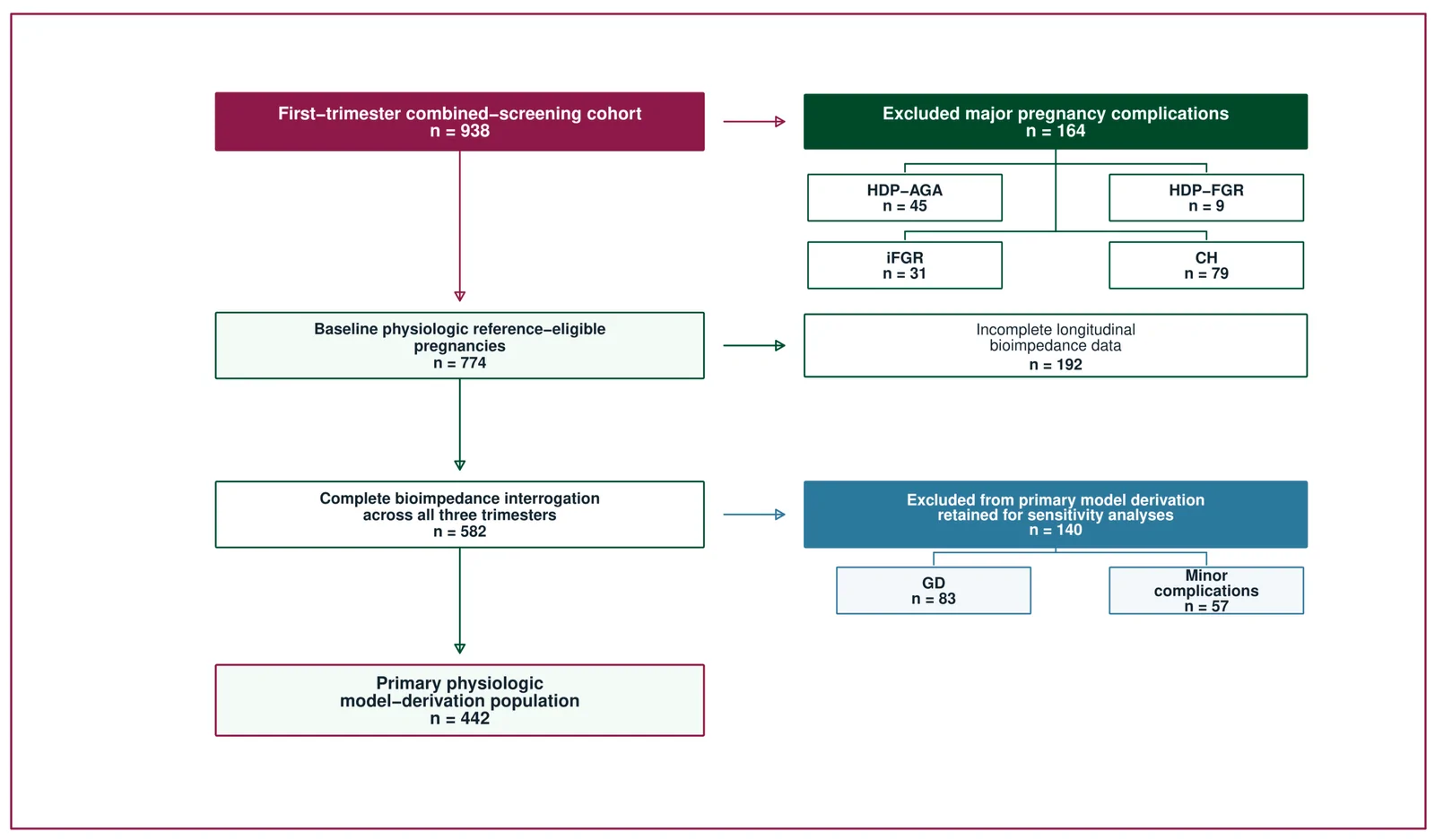
Study flow and definition of the physiologic reference population. HDP-AGA, hypertensive disorders of pregnancy with an appropriate-for-gestational-age fetus; HDP-FGR, hypertensive disorders of pregnancy with fetal growth restriction; iFGR, isolated fetal growth restriction; CH, chronic hypertension; GD, gestational diabetes.

Among these pregnancies, 582 patients (75.2%) underwent complete bioimpedance interrogations across all three trimesters. Evaluation of baseline characteristics between women with complete longitudinal visits (*n* = 582) and those with missing visits (n = 192) demonstrated no significant differences in maternal age (*p* = 0.42), baseline BMI (*p* = 0.68), or parity (*p* = 0.31). Within this longitudinal subgroup, 83 (14.3%) developed GD, 9 (1.5%) had preterm birth, 4 (0.7%) developed intrahepatic cholestasis of pregnancy, and 46 (7.9%) had other recorded minor obstetric complications. These 140 pregnancies were excluded from the primary normative pool and retained exclusively for sensitivity analyses (Supplementary Table S1). Consequently, the final cohort on which physiologic model derivation was performed included 442 pregnancies (Figure 1).

Baseline maternal characteristics, stratified by enrolling center, are detailed in Table 1. Maternal age and parity differed significantly between centers, whereas no significant between-center differences were observed in pre-pregnancy maternal height or BMI.

### 3.2. Gestational Trajectories and Reference Curves

Bioimpedance values stratified by center and gestational trimester are summarized in Table 2. Across all three clinical centers, a progressive, consistent increase from the first to the third trimester was observed for Total Body Water, Lean Mass, Fat Mass, and Visceral Fat level.

**Figure 2:**
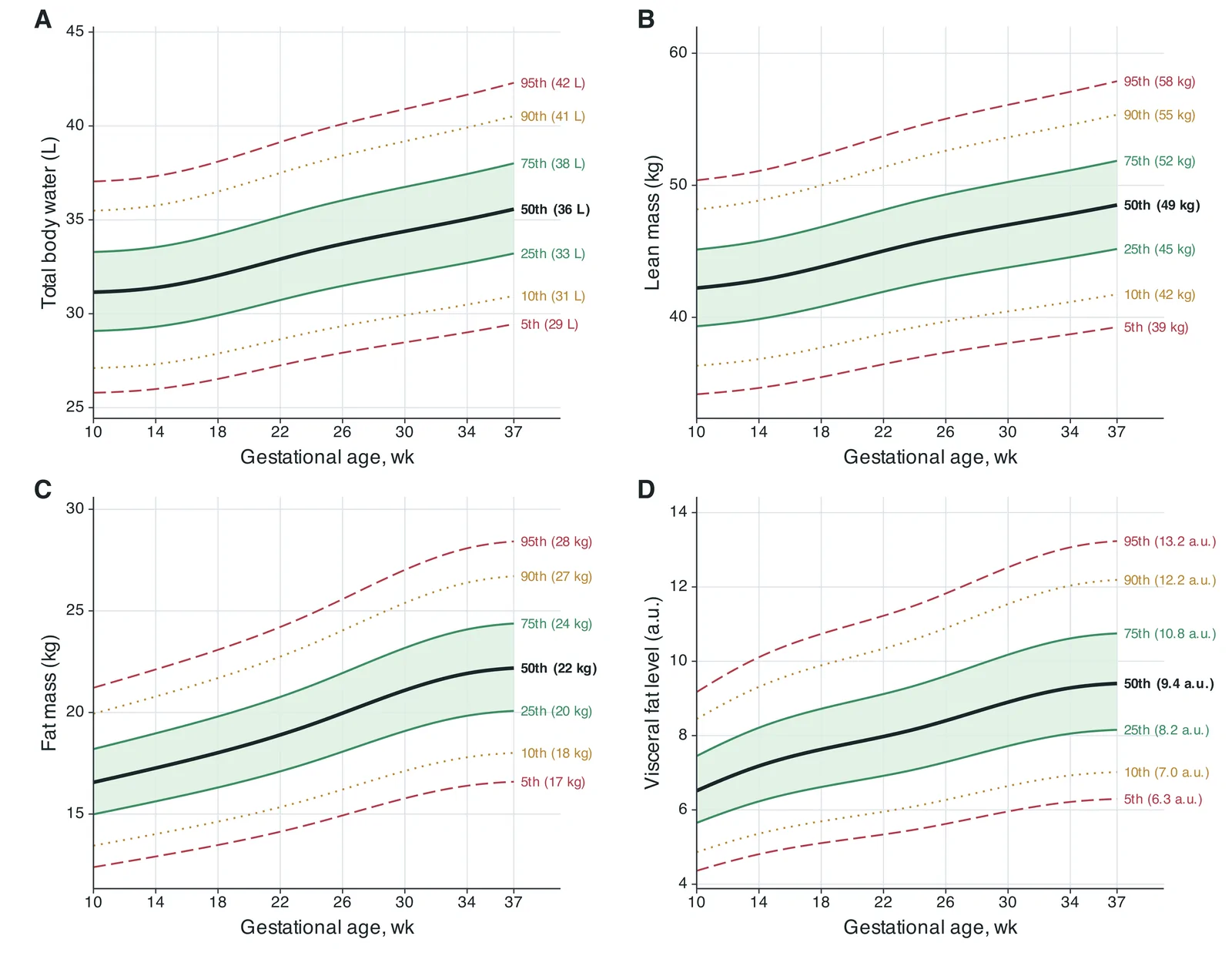
Gestational age-specific reference centile curves for maternal bioimpedance parameters across the full range of observed gestational ages (weeks 10 to 37). Reference centile curves are shown for total body water (**A**), lean mass (**B**), fat mass (**C**), and visceral fat level (**D**). Curves were estimated in the physiologic reference population for a nonsmoking patient with a pre-pregnancy BMI equal to the population median. The black line represents the estimated 50th centile. Shaded regions represent the central 50% reference interval (25th–75th centiles), central 80% reference interval (10th–90th centiles), and central 90% reference interval (5th–95th centiles). Values outside the 5th–95th centile interval are outside the fitted central reference range for the specified covariate profile.

Although recruitment was targeted around three primary gestational windows (10–15, 23–25, and 34–37 weeks), the continuous trajectories presented across gestational age represent model-based smooth interpolations linking these empirical assessment windows (Figure 2).

The mathematical details of model selection, including candidate distributional families and scale structures, are reported in Supplementary Table S2, while the final GAMLSS parameter estimates are provided in Supplementary Table S3.

To illustrate these trajectories, for a representative non-smoking patient with a pre-pregnancy BMI of 21.6 (corresponding to the median BMI of the healthy reference population), the estimated parameters progressed as follows:

- Total Body Water: Increased from 31.2 L at 12 weeks to 33.3 L at 24 weeks and 35.0 L at 34 weeks, with 90% reference intervals (5th–95th centiles) spanning 25.9 to 37.1 L, 27.6 to 39.6 L, and 29.0 to 41.7 L, respectively;
- Lean Mass: The estimated median increased from 42.5 kg (T1) to 45.6 kg (T2) and 47.8 kg (T3);
- Fat Mass: The estimated median increased from 16.9 kg (T1) to 19.4 kg (T2) and 21.9 kg (T3);
- Visceral Fat Level: The estimated median increased from 6.9 a.u. (T1) to 8.2 a.u. (T2) and 9.3 a.u. (T3).

### 3.3. BMI-Conditioned Contour Plots and Model Calibration

**Figure 3:**
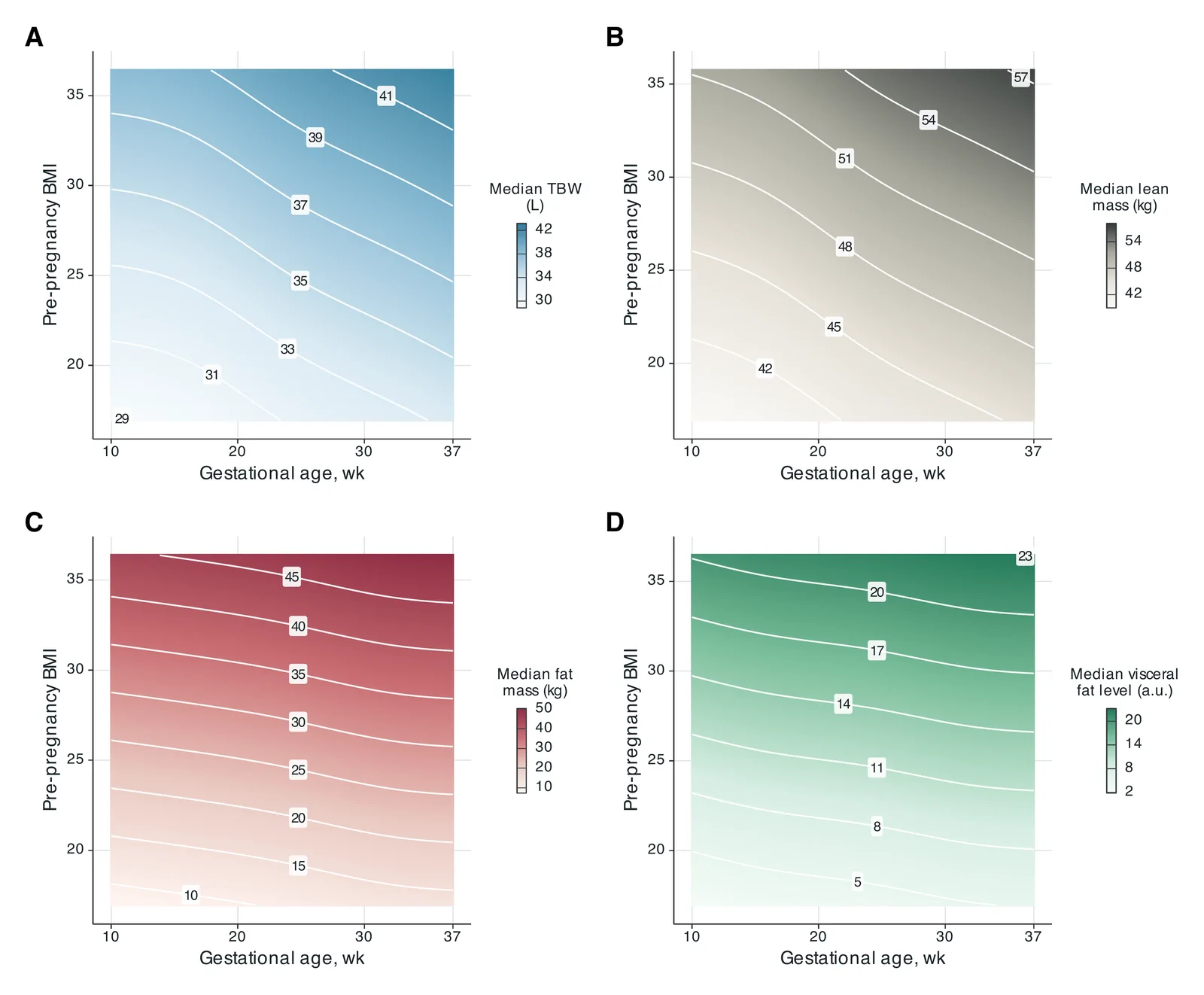
Estimated median bioimpedance values according to pre-pregnancy BMI and gestational age across the full range of observed gestational ages (weeks 10 to 37). Panels show model-estimated median values (50th centile) for total body water (**A**), lean mass (**B**), fat mass (**C**), and visceral fat level (**D**). Gestational age is shown on the *x*-axis and pre-pregnancy BMI is shown on the *y*-axis. Color shading represents the estimated median value, and labeled contour lines connect combinations with the same estimated median. Values are expressed in liters for total body water, kilograms for lean and fat mass, and arbitrary units for visceral fat level.

BMI and gestational age conditioned contour plots are presented in Figure 3. For each 1-unit increase in pre-pregnancy BMI, there was a significant increase in the estimated median of Total Body Water (estimated median of 0.47 L; 95% CI, 0.38–0.57), Lean Mass (0.63 kg; 95% CI, 0.50–0.76), Fat Mass (1.88 kg; 95% CI, 1.77–1.99), and Visceral Fat level (0.92 a.u.; 95% CI, 0.85–0.98).

The pre-pregnancy BMI gradient was modest for Total Body Water and Lean Mass, whereas it was markedly more pronounced for Fat Mass and Visceral Fat level. To facilitate reproducibility and clinical implementation, the full operational equations for calculating individualized normality values and reference limits, along with a worked clinical example, are detailed in the Supplementary Statistical Appendix. Similar associations were observed in the longitudinal sensitivity analysis (Supplementary Table S6). The corresponding reference centile curves are compared with those from the primary analysis in Supplementary Figure S2.

Model calibration and goodness-of-fit assessments are reported in Figure S1 and Table S4. Overall calibration was highly consistent with the nominal centile structure. Within the healthy reference population, the observed proportion of patients falling below the 5th centile ranged from 4.7% to 5.2% across all endpoints, while the observed proportion above the 95th centile ranged from 4.4% to 5.6%. Empirical coverage of the central 90% reference interval ranged from 89.4% to 90.8%, closely matching the theoretical 90% target. Normalized quantile residuals exhibited mean values close to 0 and standard deviations of 1.00 across all endpoints, with Q-Q plots demonstrating no relevant tail mis-calibration. These metrics reflect internal model fit within the development cohort and should not be interpreted as external validation.

Finally, center-adjusted sensitivity models did not materially alter primary model selection or calibration parameters (Table S5). All endpoints retained a Box–Cox t-distribution with a constant scale, tail calibration remained close to nominal values, and estimated center-related shifts did not alter the overall gestational and BMI-dependent architecture of the normative reference curves.

## 4. Discussion

### 4.1. Principal Findings and Originality

Our results demonstrated a steady, progressive, trimester-by-trimester increasing trajectory in Total Body Water, Lean Mass, and Fat Mass in uncomplicated healthy pregnancies. A key physiological insight emerging from our mathematical modeling was the stark contrast in how pre-pregnancy BMI influences distinct maternal body compartments. While a 1-unit increase in pre-pregnancy BMI exerted a minimal, tightly calibrated effect on TBW and Lean Mass, it demonstrated a profound, disproportionate impact on maternal Fat Mass and Visceral Fat trajectories.

To the best of our knowledge, this was the first multicenter, prospective study to establish longitudinal, trimester-specific bioelectrical impedance reference curves tailored exclusively to a large, strictly selected cohort of physiologically uncomplicated pregnancies.

While previous obstetric studies had utilized bioelectrical impedance analysis [18,19], they routinely relied on reference equations or vectors calibrated on non-pregnant populations, which inherently fail to account for the unique, fluid-driven, and metabolic dynamics of gestation. By applying generalized additive models for location, scale, and shape (GAMLSS) and utilizing BMI-conditioned contour plots, our work provided a clinically readable benchmark that integrated both gestational age and pre-pregnancy maternal BMI.

The trajectories observed in this large cohort aligned with the macro-physiological adaptations that are well-documented in classic obstetric literature, such as those pioneered by Hytten [1] and Lukaski [21], which track progressive hemodilution and maternal-fetal tissue expansion. However, by excluding pregnancies that subsequently developed even mild disorders (such as gestational diabetes or late-onset cholestasis), our final model-derivation population represented a highly filtered reference cohort of true “normality.” Compared to prior longitudinal BIA studies in healthy pregnant cohorts, which were often limited by small single-center sample sizes or lacked rigorous post hoc complication screening [25,26,27], our multicenter design and strict exclusion criteria ensured that these curves reflected a highly reliable physiological baseline.

### 4.2. Maternal Hydration Stability vs. Adipose-Driven Meta-Inflammation

These biologically diverging trajectories between TBW and Lean Mass versus the impact of BMI on Fat Mass and Visceral Fat carry significant clinical implications. The remarkable stability of the TBW trajectory across different pre-pregnancy BMI strata suggested that gestational fluid expansion is a highly preserved, “hardwired” evolutionary adaptation designed to support placental perfusion [1,2,3]. In uncomplicated pregnancies, the cardiovascular and renal remodeling required to expand plasma volume operates within strict physiological boundaries, largely shielded from baseline maternal adiposity [2,22]. Consequently, our stable TBW reference curves provide a universal baseline framework to describe maternal hydration trends. Deviations above the 95th centile describe statistical non-conformity relative to a physiological cohort, presenting a hypothesis-generating marker that requires prospective testing in cohorts with fluid overload or hypertensive complications [2,28].

In contrast, the high sensitivity of Fat Mass and Visceral Fat to pre-pregnancy BMI highlighted these compartments as dynamic, highly variable parameters. In the context of maternal-fetal medicine, this sensitivity is far from benign. Excess visceral adiposity does not merely represent a passive energy reserve; rather, it acts as an active immunometabolic organ [8,9,10]. Hypertrophic visceral adipose tissue is a primary driver of chronic, low-grade meta-inflammation, characterized by an accelerated secretion of pro-inflammatory cytokines (such as TNF-alpha and IL-6) and a concomitant reduction in protective adiponectin [9,11,12]. This persistent inflammatory cascade promotes systemic insulin resistance and endothelial dysfunction, directly predisposing patients to gestational diabetes and hypertensive disorders [13,15,16]. By demonstrating that Fat Mass and Visceral Fat trajectories were highly dependent on baseline BMI, our study provided clinicians with a sensitive tool to monitor these inflammation-prone compartments.

### 4.3. Clinical Applications

These reference curves offer three immediate clinical utilities:

1. Hypothesis Framework for Metabolic Research: Tracking a patient’s Fat Mass and Visceral Fat trajectories on our BMI-conditioned contour plots allows clinicians to identify women deviating above the 95th centile. This serves as an early, subclinical proxy for active meta-inflammation, signaling the need for timely, personalized nutritional and lifestyle interventions before systemic vascular or metabolic damage occurs [7,8].
2. Evaluation of Fluid Disturbances: In disorders like preeclampsia, altered expression of angiogenic factors induces capillary leakage [15]. This vascular damage manifests as “occult edema,” which occurs weeks before the onset of clinical hypertension or overt proteinuria. Utilizing our normative TBW centiles as a baseline benchmark will enable clinicians to identify sudden, abnormal fluid shifts, serving as a valuable asset for early risk-stratification of hypertensive phenotypes [26,27,29].
3. Monitoring Nutritional Trends: Monitoring Lean Mass and TBW trajectories ensures that therapeutic dietary restrictions, often imposed on patients with rapid weight gain, do not inadvertently induce maternal malnutrition, poor hydration, or compromise fetal growth [5,6,7,8,9,10,11,12,13].

### 4.4. Strengths and Limitations

This study has some limitations. First, the study population was primarily composed of Italian women of Caucasian ancestry, which limits direct generalizability to other ethnic populations with distinct baseline body composition profiles. Second, pregnancy itself limits the possible technologies that can be adopted. Therefore, body composition parameters were estimated using the built-in multi-frequency algorithms of a commercial standing BIA device (InBody 270), rather than direct reference methods (e.g., isotopic dilution techniques or DXA); further studies should confirm alignment with supine tetrapolar BIA systems. Third, BIA assessments were obtained at three discrete gestational windows (10–15, 23–25, and 34–37 weeks); consequently, the continuous centile lines represent model-based smooth interpolations and cannot capture short-term or acute temporal fluid fluctuations. Fourth, the multi-step post-hoc filtering (which reduced the cohort from 938 (enrolled) to 442 (analyzed)) was necessary to establish a strictly uncomplicated reference pool, but could introduce minor selection attrition bias. Finally, model calibration metrics reflect internal goodness-of-fit within the development cohort; external validation in independent international cohorts is still required.

Despite these limitations, the multicenter design and broadly consistent findings from GAMLSS and longitudinal sensitivity analyses support the derived curves as a reference framework for maternal bioimpedance in uncomplicated pregnancy, although external validation remains necessary before clinical application.

## 5. Conclusions

This study provides multicenter, BMI-conditioned, trimester-specific reference ranges for maternal bioimpedance parameters in uncomplicated pregnancies. These normative benchmarks could support the identification of deviations in maternal hydration and body composition and facilitate future research on personalized nutritional and obstetric monitoring.

## Figures and Tables

**Table 1:** Baseline characteristics of the cohort by center.

Characteristic	Overall	Mangiagalli	Monza	Trieste	*p* Value
No. of pregnancies	442	300	106	36	
***Maternal*** ***characteristics***					
Age, y	33.0 (31.0–36.0)	34.0 (31.0–36.0)	33.0 (30.0–35.0)	32.5 (29.8–34.2)	0.003
Pre-pregnancy BMI	21.6 (20.3–24.4)	21.5 (20.2–24.1)	22.4 (20.3–24.9)	22.1 (21.0–24.7)	0.200
Height, cm	165.0 (160.0–169.0)	165.0 (160.0–170.0)	163.0 (160.0–168.0)	163.0 (160.0–169.2)	0.239
Pre-pregnancy weight, kg	60.0 (54.0–66.9)	59.1 (54.0–65.5)	60.0 (55.0–67.0)	60.0 (55.8–67.0)	0.565
Gestational weight gain, kg	12.0 (10.0–15.0)	11.0 (9.4–14.0)	13.0 (10.0–15.2)	12.0 (11.0–15.0)	0.104
***Smoking status***					
Pre-pregnancy smoking	106 (24.0)	83 (27.7)	20 (18.9)	3 (8.3)	0.011
Active smoking during pregnancy	19 (4.3)	12 (4.0)	5 (4.8)	2 (5.6)	0.798
***Obstetric history***					
Nulliparous	322 (72.9)	216 (72.0)	96 (90.6)	10 (27.8)	<0.001
***Delivery and neonatal outcomes***					
BMI at delivery	26.4 (24.6–29.4)	26.2 (24.5–28.7)	26.6 (24.9–30.8)	27.1 (25.8–29.4)	0.194
Gestational age at delivery, wk	39.7 (39.0–40.6)	39.4 (38.9–40.4)	40.1 (39.3–40.9)	40.1 (39.5–41.0)	0.004
Neonatal birth weight, g	3285 (3035–3540)	3325 (3064–3530)	3160 (2940–3540)	3310 (3148–3552)	0.122

Values are median (interquartile range) or number (%). *p* values were calculated using Kruskal–Wallis tests for continuous variables and Fisher’s exact tests for categorical variables.

**Table 2:** Bioimpedance values by center and trimester in the physiologic reference population.

Visit	Overall	Mangiagalli	Monza	Trieste	*p* Value
***Total body water (L)***					
T1	31.8 (29.8–34.5)	32.0 (29.9–34.6)	31.2 (28.9–33.4)	33.1 (30.4–36.2)	0.020
T2	34.0 (31.2–36.6)	34.2 (31.5–36.8)	33.1 (30.7–35.5)	34.7 (31.0–38.1)	0.073
T3	35.5 (33.1–38.4)	35.5 (33.3–38.5)	35.1 (32.6–37.3)	37.7 (33.3–39.6)	0.187
***Lean mass (kg)***					
T1	43.4 (40.5–47.1)	43.8 (40.8–47.4)	42.0 (38.7–45.3)	45.4 (41.5–49.5)	0.001
T2	46.5 (42.5–50.1)	46.8 (43.2–50.2)	45.1 (41.9–48.4)	47.5 (42.5–52.4)	0.019
T3	48.3 (45.1–52.3)	48.5 (45.3–52.6)	47.8 (43.8–50.7)	51.9 (46.0–54.7)	0.037
***Fat mass (kg)***					
T1	17.7 (14.2–23.8)	17.4 (13.8–23.1)	18.9 (15.0–24.3)	16.8 (15.1–23.2)	0.448
T2	20.1 (16.3–25.3)	19.6 (16.2–25.0)	21.6 (17.9–26.3)	19.2 (16.4–24.5)	0.143
T3	22.7 (18.6–27.9)	22.2 (18.4–27.6)	24.0 (19.7–29.2)	21.6 (18.0–27.0)	0.136
***Visceral fat level (a.u.)***					
T1	7.0 (5.0–11.0)	7.0 (5.0–11.0)	8.0 (6.0–11.0)	6.0 (5.0–9.2)	0.329
T2	8.0 (6.8–12.0)	8.0 (6.0–11.2)	9.0 (7.0–12.0)	8.0 (6.2–10.0)	0.296
T3	10.0 (7.0–13.0)	10.0 (7.0–13.0)	10.0 (8.0–14.0)	9.0 (7.0–12.0)	0.214

Values are median (interquartile range). *p* values were calculated using Kruskal–Wallis tests. The table includes centers with valid bioimpedance measurements contributing to the primary reference models. TBW, total body water; T1, first trimester; T2, second trimester; T3, third trimester.

## Data Availability

The data that support the findings of this study are available from the corresponding author upon reasonable request.

## Supplementary Materials

The following supporting information can be downloaded at: https://www.mdpi.com/article/10.3390/nu18203341/s1, Figure S1: Calibration of the Bioimpedance Reference Models; Figure S2. Comparison of GAMLSS and longitudinal bioimpedance reference curves; Table S1. Baseline characteristics of the broader population cohort stratified by center; Table S2. Candidate distributional models for the reference curves; Table S3. Primary GAMLSS models and covariate effects; Table S4. Model calibration; Table S5. Sensitivity analyses; Table S6. Longitudinal models and covariate effects; and a Statistical Appendix containing the operational reference equations and a worked example.

## Abbreviations

BIA, bioelectrical impedance analysis; BIC, Bayesian information criterion; BMI, body mass index; CH, chronic hypertension; CI, confidence interval; FM, fat mass; FGR, fetal growth restriction; GAMLSS, generalized additive models for location, scale, and shape; GD, gestational diabetes; GWG, gestational weight gain; HDP, hypertensive disorders of pregnancy; IQR, interquartile range; LM, lean mass; PTB, preterm birth; TBW, total body water; VF, visceral fat level.

## References

[1] Hytten F. (1985). Blood volume changes in normal pregnancy. Clin. Haematol..

[2] Valensise H., Andreoli A., Lello S., Magnani F., Romanini C., De Lorenzo A. (2000). Multifrequency bioelectrical impedance analysis in women with a normal and hypertensive pregnancy. Am. J. Clin. Nutr..

[3] Ståhlberg A., Gustafsson C.K., Engtröm K., Thomsen C., Dolatabadi S., Jonasson E., Li C.-Y., Ruff D., Chen S.-M., Åman P. (2014). Hypertensive disorders of pregnancy: A systematic review of fluid and hemodynamic changes. PLoS ONE.

[4] Magee L.A., Brown M.A., Hall D.R., Gupte S., Hennessy A., Karumanchi S.A., Kenny L.C., McCarthy F., Myers J., Poon L.C. (2022). The 2021 International Society for the Study of Hypertension in Pregnancy classification, diagnosis & management recommendations for international practice. Pregnancy Hypertens..

[5] Fatima Y., Al Mamun A., King J.C. (2021). Fetal programming and the impact of maternal body composition on international benchmarks of infant growth. Am. J. Clin. Nutr..

[6] Goldstein R.F., Abell S.K., Ranasinha S., Misso M., Boyle J.A., Black M.H., Li N., Hu G., Corrado F., Rode L. (2017). Association of gestational weight gain with maternal and infant outcomes: A systematic review and meta-analysis. JAMA.

[7] Poston L., Caleyachetty R., Cnattingius S., Corvalán C., Uauy R., Herring S., Gillman M.W. (2016). Preconceptional and maternal obesity: Epidemiology and health consequences. Lancet Diabetes Endocrinol..

[8] Catalano P.M., Shankar K. (2017). Obesity and pregnancy: Mechanisms of short term and long term adverse consequences for mother and child. BMJ.

[9] Hotamisligil G.S. (2017). Foundations of immunometabolism and metainflammation. Nature.

[10] Kirwan J.P., Hauguel-De Mouzon S., Lepercq J., Challier J.C., Huston-Presley L., Friedman J.E., Kalhan S.C., Catalano P.M. (2002). TNF-alpha is a predictor of insulin resistance in human pregnancy. Diabetes.

[11] Gibney J., Healy M.-L., Smith T.P., McKenna T.J. (2008). Obesity in pregnancy stimulates macrophage activation and inflammation in the placenta. J. Clin. Endocrinol. Metab..

[12] Zembala-Szczerba M., Jaworowski A., Huras H., Babczyk D., Jach R. (2017). Low-Grade Metabolically-Induced Inflammation Mediators Interleukin-6, Adiponectin, and TNF-α Serum Levels in Obese Pregnant Patients in the Perinatal Period. Med. Sci. Monit. Basic Res..

[13] Jansson T., Powell T.L. (2007). Placental nutrient transport and fetal growth for gestational age. Int. J. Obes..

[14] Pantham P., Aye I.L., Powell T.L. (2015). Review: The role of mitochondrial dysfunction and elevated cytokines in the pathogenesis of preeclampsia and gestational diabetes. Placenta.

[15] Maynard S.E., Min J.-Y., Merchan J., Lim K.-H., Li J., Mondal S., Libermann T.A., Morgan J.P., Sellke F.W., Stillman I.E. (2003). Excess placental soluble fms-like tyrosine kinase 1 (sFlt1) may contribute to endothelial dysfunction in preeclampsia. J. Clin. Investig..

[16] McIntyre H.D., Catalano P., Zhang C., Desoye G., Mathiesen E.R., Damm P. (2019). Gestational diabetes mellitus. Nat. Rev. Dis. Primers.

[17] Moroni A., Vardè C., Giustetto A., Stagi S., Marini E., Micheletti Cremasco M. (2022). Bioelectrical Impedance Vector Analysis (BIVA) for the monitoring of body composition in pregnancy. Eur. J. Clin. Nutr..

[18] Widen E.M., Gallagher D. (2014). Body composition changes in pregnancy: Measurement, predictors and outcomes. Eur. J. Clin. Nutr..

[19] Sarno M., Di Maira T., Valensise H. (2021). Maternal body composition changes in pregnancy tracked by bioelectrical impedance analysis: A systematic review. J. Matern Fetal Neonatal Med..

[20] Staelens A.S., Vonck S., Gyselaers W. (2016). Bioelectrical impedance analysis in pregnant women: A comparison of dynamic changes in body composition. Physiol. Meas..

[21] Lukaski H.C., Siders W.A., Nielsen E.J., Hall C.B. (1994). Total body water in pregnancy: Assessment by using bioelectrical impedance. Am. J. Clin. Nutr..

[22] Ward L.C. (2019). Bioelectrical impedance analysis for body composition assessment: Reflections on accuracy, clinical utility, and standardisation. Eur. J. Clin. Nutr..

[23] Lukaski H.C., Piccoli A., Kyle U.G. (2019). Refinement of bioelectrical impedance vector analysis (BIVA) to appreciate tissue hydration and cellularity in human health and disease. Nutrients.

[24] https://uk.inbody.com/wp-content/uploads/2019/01/270_manual_ENG_C_170714.pdf.

[25] Piuri G., Ferrazzi E., Bulfoni C., Mastricci L., Di Martino D., Speciani A.F. (2017). Longitudinal changes and correlations of bioimpedance and anthropometric measurements in pregnancy: Simple possible bed-side tools to assess pregnancy evolution. J. Matern Fetal Neonatal Med..

[26] Larciprete G., Valensise H., Vasapollo B., Altomare F., Sorge R., Casalino B., De Lorenzo A., Arduini D. (2003). Body composition during normal pregnancy: Reference ranges. Acta Diabetol..

[27] Bosaeus M., Andersson-Hall U., Andersson L., Karlsson T., Ellegård L., Holmäng A. (2020). Body Composition During Pregnancy: Longitudinal Changes and Method Comparisons. Reprod. Sci..

[28] Staelens A.S., Vonck S., Molenberghs G., Malbrain M.L., Gyselaers W. (2016). Maternal body fluid composition in uncomplicated pregnancies and preeclampsia: A bioelectrical impedance analysis. Eur. J. Obstet. Gynecol. Reprod. Biol..

[29] Di Martino D.D., Sabattini E., Parasiliti M., Viscioni L., Zaccone E., Cerri S., Tinè G., Ferrazzi E. (2025). Exploring new predictors for hypertensive disorders of pregnancy. Best Pract. Res. Clin. Obstet. Gynaecol..

