# Supplementary material for: Trimester-Specific Bioelectrical Impedance Reference Curves in Physiological Pregnancy: Mapping Maternal Body Composition and Hydration Trajectories
Source: Nutrients. 2026 Oct 12;18(20):3341. doi: 10.3390/nu18203341 (PMC13708494; doi:10.3390/nu18203341)
Supplement: Supplementary file 1 [file nutrients-18-03341-s001.zip › nutrients-4564764-supplementary.pdf]

MDPI

nutrients

Supplementary Materials

Trimester-Specific Bioelectrical Impedance Reference Curves in Physiological Pregnancy: Mapping Maternal Body Composition and Hydration Trajectories

Statistical Appendix

Operational equations for calculating individualized gestational age- and BMI-conditioned reference values and central 90% reference intervals.

Statistical Appendix

These equations describe the primary gestational age-specific reference centile curves for maternal bioimpedance parameters between 12 and 40 weeks.

Let GA be gestational age in weeks, BMI pre-pregnancy body mass index in kg/m^2, and $S = 1$ for pre-pregnancy smoking and $S = 0$ otherwise.

Calculation steps

- **Step 1.** Select the bioimpedance parameter.
- **Step 2.** Select the row corresponding to the patient's gestational age.
- **Step 3.** Compute Δ, defined in the selected row.
- **Step 4.** Calculate the median μ, then multiply μ by the 5th and 95th centile multipliers.

$$\mu = a_0 + a_1\Delta + a_2\Delta^2 + a_3\Delta^3 + \beta_{\mathrm{BMI}}\mathrm{BMI} + \beta_S S$$
$$C_5 = m_5\mu, \qquad C_{50} = \mu, \qquad C_{95} = m_{95}\mu$$

The 5th and 95th centiles define the central 90% physiologic reference interval. Values below C_5 or above C_{95} are outside the fitted reference range.

Piecewise equations for calculation of bioimpedance reference centiles

Each box gives the median equation and interval-specific coefficients. Use only the row corresponding to the patient's gestational age. TBW indicates total body water; LM, lean mass; FM, fat mass; VF, visceral fat level.

Total body water (L)

$$\mu_{\mathrm{TBW}} = a_0 + a_1\Delta + a_2\Delta^2 + a_3\Delta^3 + 0.47\,\mathrm{BMI} + 0.34\,S$$

GA, wk	Δ	a_0	a_1	a_2	a_3
12–<14	$GA-12$	21.0	0.06	0.011	0.0009
14–<24	$GA-14$	21.1	0.11	0.016	-0.0008
24–<34	$GA-24$	23.1	0.20	-0.007	0.0003
34–40	$GA-34$	24.8	0.17	0.003	-0.0001

$$C_5 = 0.83\mu \quad C_{50} = \mu \quad C_{95} = 1.19\mu$$

Lean mass (kg)

$$\mu_{\mathrm{LM}} = a_0 + a_1\Delta + a_2\Delta^2 + a_3\Delta^3 + 0.63\,\mathrm{BMI} + 0.44\,S$$

GA, wk	Δ	a_0	a_1	a_2	a_3
12–<14	$GA-12$	28.8	0.14	0.011	0.0009
14–<24	$GA-14$	29.1	0.20	0.017	-0.0009
24–<34	$GA-24$	31.9	0.27	-0.009	0.0004
34–40	$GA-34$	34.1	0.21	0.003	-0.0001

$$C_5 = 0.81\mu \quad C_{50} = \mu \quad C_{95} = 1.19\mu$$

Fat mass (kg)

$$\mu_{\mathrm{FM}} = a_0 + a_1\Delta + a_2\Delta^2 + a_3\Delta^3 + 1.88\,\mathrm{BMI} + 0.47\,S$$

GA, wk	Δ	a_0	a_1	a_2	a_3
12–<14	$GA-12$	-23.7	0.18	0.001	0.0001
14–<24	$GA-14$	-23.4	0.18	0.001	0.0002
24–<34	$GA-24$	-21.2	0.27	0.008	-0.0009
34–40	$GA-34$	-18.7	0.14	-0.020	0.0008

$$C_5 = 0.75\mu \quad C_{50} = \mu \quad C_{95} = 1.28\mu$$

Visceral fat level (a.u.)

$$\mu_{\mathrm{VF}} = a_0 + a_1\Delta + a_2\Delta^2 + a_3\Delta^3 + 0.92\,\mathrm{BMI} + 0.23\,S$$

GA, wk	Δ	a_0	a_1	a_2	a_3
12–<14	$GA-12$	-13.0	0.17	-0.006	-0.0005
14–<24	$GA-14$	-12.7	0.14	-0.009	0.0005
24–<34	$GA-24$	-11.7	0.11	0.006	-0.0005
34–40	$GA-34$	-10.6	0.07	-0.010	0.0004

$$C_5 = 0.67\mu \quad C_{50} = \mu \quad C_{95} = 1.41\mu$$

Worked example for applying the reference equations

Illustrative profile: 28.0 gestational weeks, pre-pregnancy BMI 21.6 kg/m^2, nonsmoker.

At 28 weeks, select the 24 to <34 weeks row. Therefore $\Delta = GA - 24 = 4$. Smoking is coded $S = 0$.

Step 2. Calculate the median and reference limits. For example, fat mass is calculated as:

$$\mu_{\mathrm{FM}} = -21.25 + 0.269(4) + 0.0075(4^2) - 0.00093(4^3) + 1.88(21.6) + 0.47(0)$$
$$= \mathbf{20.5}\,\mathrm{kg}$$

$$C_{5,\mathrm{FM}} = 0.75 \times 20.5 = \mathbf{15.4}\,\mathrm{kg}$$
$$C_{95,\mathrm{FM}} = 1.28 \times 20.5 = \mathbf{26.3}\,\mathrm{kg}$$
$$\mathrm{Observed} = \mathbf{31.0}\,\mathrm{kg} > 26.3\,\mathrm{kg} \quad \Rightarrow \quad \textbf{Above 95th}$$

Step 3. Apply the same rule to all 4 parameters.

**Parameter**	μ	C_5	C_{95}	**Observed**	**Interpretation**
Total body water	34.1	28.2	40.5	34.0	**Within range**
Lean mass	46.6	37.7	55.6	46.5	**Within range**
Fat mass	20.5	15.4	26.3	31.0	**Above 95th**
Visceral fat level	8.7	5.8	12.2	14.0	**Above 95th**

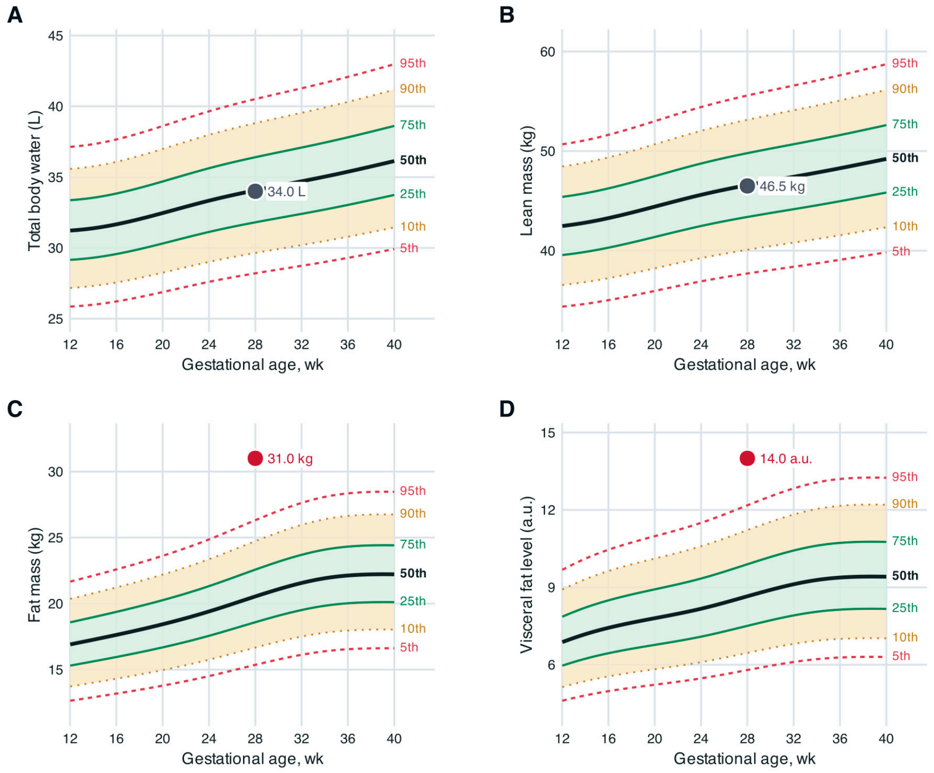

The red points identify measurements above the fitted 95th centile. Values inside the 5th–95th interval are shown in gray.

Supplementary Figure

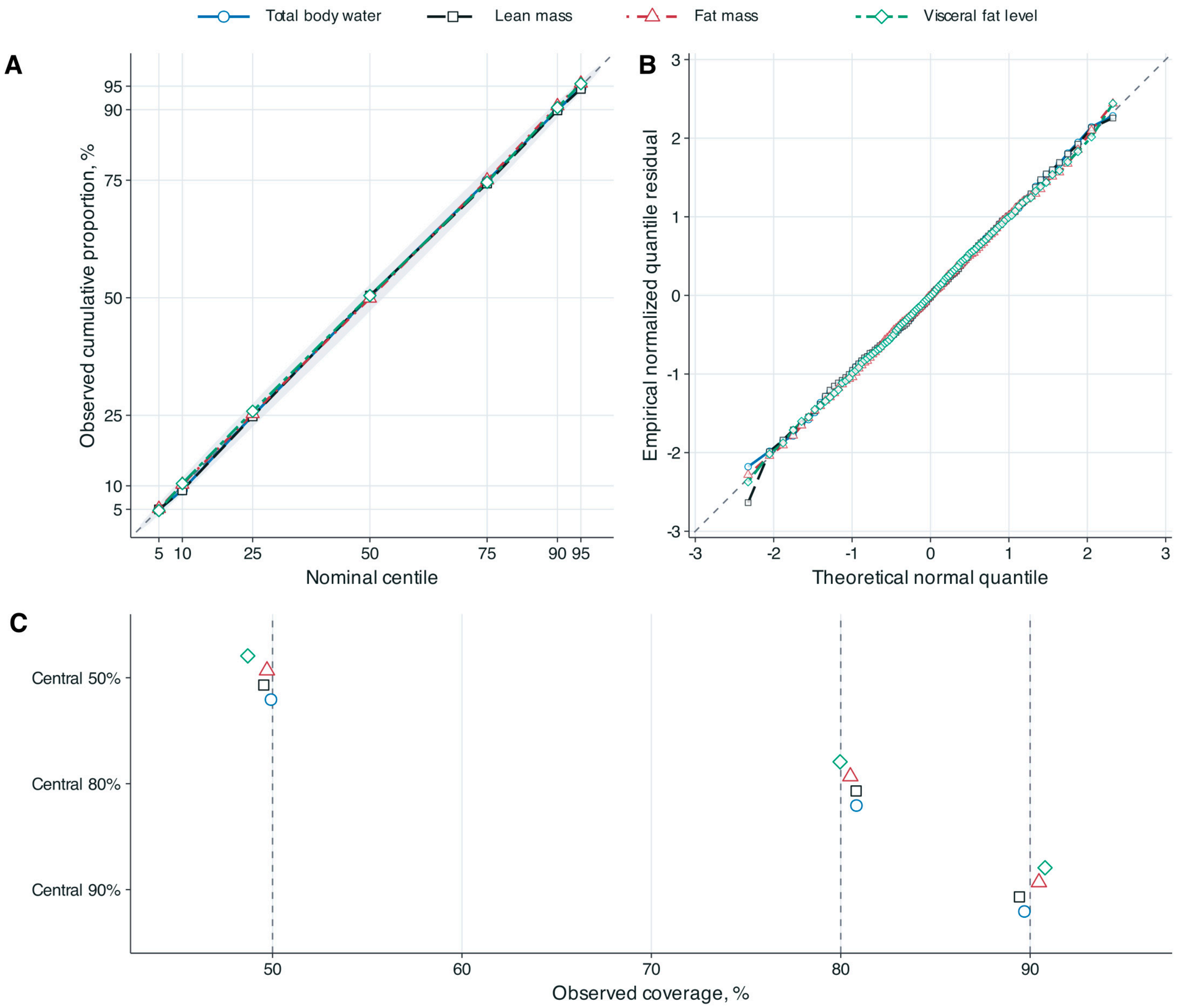

Figure S1. Calibration of the bioimpedance reference models. Panel A shows observed cumulative proportions against nominal centiles; the dashed diagonal indicates perfect calibration. Panel B shows Q–Q plots of normalized quantile residuals against theoretical standard normal quantiles. Panel C shows empirical coverage of the central 50%, 80%, and 90% reference intervals; vertical dashed lines indicate nominal coverage.

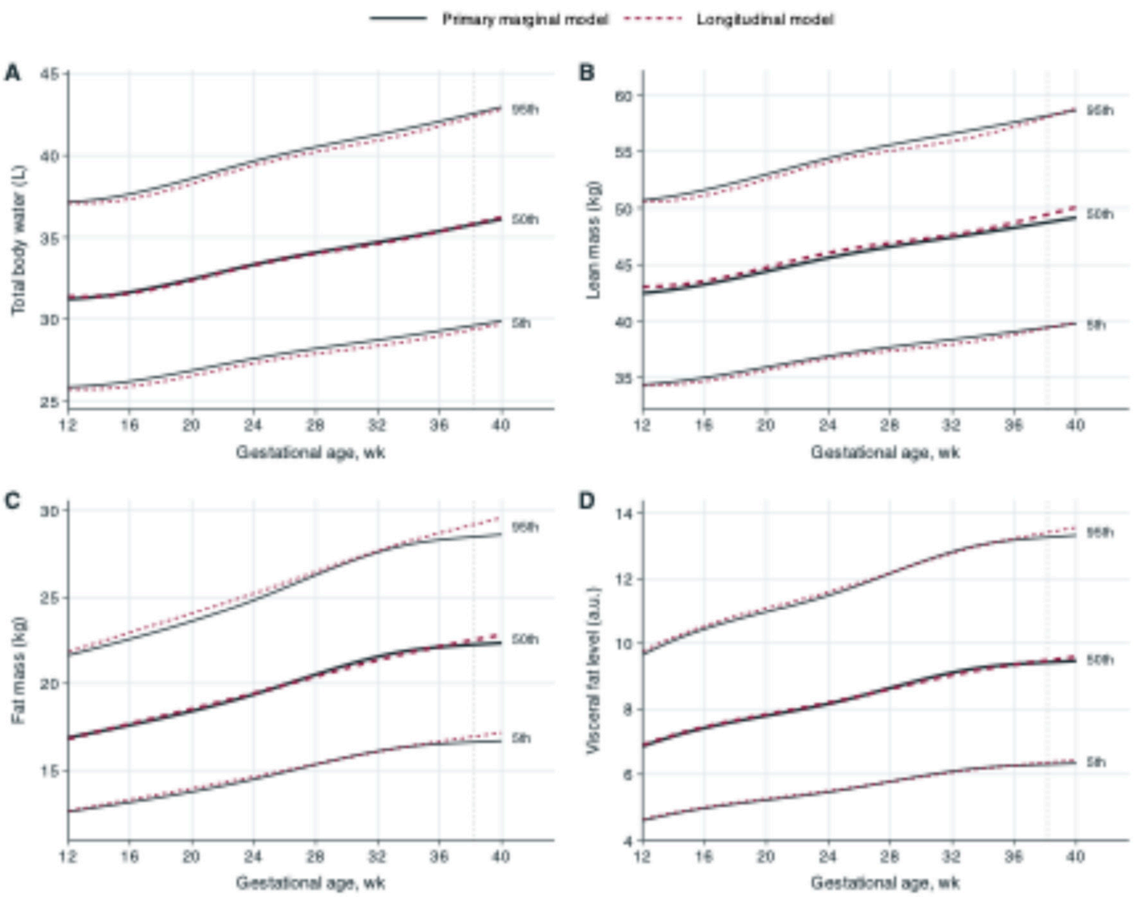

Figure S2. Comparison of GAMLSS and longitudinal bioimpedance reference curves. Gestational age-specific 5th, 50th, and 95th centiles are shown for total body water (**A**), lean mass (**B**), fat mass (**C**), and visceral fat level (**D**), for a nonsmoking patient with pre-pregnancy BMI 21.6. Charcoal solid curves represent the primary marginal models; maroon dashed curves represent longitudinal models with the same marginal specifications and an unstructured Gaussian-copula correlation across gestational periods. Both approaches used the same reference population, covariates, and gestational-age spline basis. Heavier curves denote the 50th centile; thinner curves denote the 5th and 95th centiles, which delimit the central 90% reference interval, not a confidence interval. The vertical gray dotted line marks the maximum gestational age in the fitting data, approximately 38.1 weeks.

Supplementary Tables

Table S1. Baseline characteristics of the broader population cohort stratified by center.

Characteristic	Mangiagalli	Monza	Trieste	P value
No. of pregnancies	407	127	48	
Maternal characteristics				
Age, y	34.0 (31.0-37.0)	33.0 (30.0-35.0)	32.0 (29.8-35.0)	<.001
Pre-pregnancy BMI	21.6 (20.2-24.2)	22.7 (20.3-25.2)	22.4 (21.0-24.9)	.089
Height, cm	165.0 (160.0-170.0)	163.0 (160.0-168.0)	164.0 (159.8-168.2)	.338
Pre-pregnancy weight, kg	59.0 (54.0-65.2)	60.0 (55.0-67.5)	60.0 (55.8-69.2)	.273
Smoking status				
Pre-pregnancy smoking	116 (28.5)	22 (17.3)	3 (6.2)	<.001
Active smoking	16 (3.9)	5 (4.0)	2 (4.2)	1.000
Obstetric history and sensitivity status				
Nulliparous women	286 (70.3)	114 (89.8)	11 (22.9)	<.001
Gestational diabetes	61 (15.0)	13 (11.0)	9 (19.1)	.347
Other minor obstetric complication	107 (28.9)	21 (19.1)	12 (30.0)	.101

Values are median (interquartile range) or number (%). P values were calculated using Kruskal–Wallis tests for continuous variables and Fisher's exact tests for categorical variables.

Table S2. Candidate distributional models for the reference curves.

Endpoint	Distribution	Scale model	BIC	Delta BIC
Fat mass				
	Box-Cox t	Constant	6282.73	0.00
	Box-Cox power exponential	Constant	6300.17	17.44
	Box-Cox t	Gestational age spline	6302.48	19.75
	Box-Cox power exponential	Gestational age spline	6317.99	35.26
	Normal	Gestational age spline	6373.18	90.45
	Box-Cox Cole-Green	Constant	6375.25	92.52
	Box-Cox Cole-Green	Gestational age spline	6385.20	102.47
	Normal	Constant	6390.13	107.41
Lean mass				
	Box-Cox t	Constant	7415.68	0.00
	Box-Cox power exponential	Constant	7428.55	12.87
	Box-Cox t	Gestational age spline	7435.73	20.04
	Box-Cox power exponential	Gestational age spline	7448.73	33.05
	Box-Cox Cole-Green	Constant	7494.36	78.68
	Box-Cox Cole-Green	Gestational age spline	7514.81	99.13
	Normal	Constant	7516.83	101.15
	Normal	Gestational age spline	7533.18	117.50
Total body water				
	Box-Cox t	Constant	6540.87	0.00
	Box-Cox power exponential	Constant	6558.40	17.52
	Box-Cox t	Gestational age spline	6561.17	20.29
	Box-Cox power exponential	Gestational age spline	6578.24	37.37
	Box-Cox Cole-Green	Constant	6597.77	56.90
	Box-Cox Cole-Green	Gestational age spline	6615.44	74.57
	Normal	Constant	6630.72	89.85
	Normal	Gestational age spline	6632.84	91.97
Visceral fat level				
	Box-Cox t	Constant	4973.07	0.00
	Box-Cox power exponential	Constant	4986.33	13.27
	Box-Cox t	Gestational age spline	4993.65	20.58
	Box-Cox power exponential	Gestational age spline	5006.65	33.58
	Box-Cox Cole-Green	Constant	5014.98	41.92
	Box-Cox Cole-Green	Gestational age spline	5034.46	61.40
	Normal	Gestational age spline	5122.37	149.30
	Normal	Constant	5133.66	160.60

Candidate models are shown for the primary physiologic model-derivation population. Delta BIC was calculated within endpoint; the selected model was the converged candidate with the lowest BIC. BIC, Bayesian information criterion.

Table S3. Primary GAMLSS models and covariate effects.

Endpoint	Location covariate	Estimate	95% CI	P value
Total body water				
	Pre-pregnancy BMI, per 1-unit increase	0.47	0.38 to 0.57	<.001
	Pre-pregnancy smoking: yes vs no	0.34	-0.50 to 1.17	.430
Lean mass				
	Pre-pregnancy BMI, per 1-unit increase	0.63	0.50 to 0.76	<.001
	Pre-pregnancy smoking: yes vs no	0.44	-0.69 to 1.56	.446
Fat mass				
	Pre-pregnancy BMI, per 1-unit increase	1.88	1.77 to 1.99	<.001
	Pre-pregnancy smoking: yes vs no	0.47	-0.21 to 1.15	.172
Visceral fat level				
	Pre-pregnancy BMI, per 1-unit increase	0.92	0.85 to 0.98	<.001
	Pre-pregnancy smoking: yes vs no	0.23	-0.12 to 0.58	.193

Estimates are from the location parameter of the selected GAMLSS model and are reported on the response scale. To account for within-participant correlation, 95% confidence intervals and two-sided P values were calculated using participant-level bootstrap standard errors from 1000 resamples stratified by recruitment center, with all measurements from each woman resampled together.

Table S4. Model calibration.

Endpoint	Level	Stratum	z mean	z SD	<5th, %	5th-95th, %	>95th, %
Fat mass							
	Overall	All	0.00	1.00	5.2	90.5	4.4
Lean mass							
	Overall	All	0.00	1.00	5.0	89.4	5.6
Total body water							
	Overall	All	0.00	1.00	4.9	89.7	5.4
Visceral fat level							
	Overall	All	0.00	1.00	4.7	90.8	4.5
Fat mass							
	Gestational age	14-27 wk	-0.03	0.98	4.9	91.5	3.6
	Gestational age	8-13 wk	0.09	1.05	4.3	89.5	6.2
	Gestational age	>=28 wk	-0.02	1.00	6.2	89.5	4.3
	Prepregnancy BMI	Lower BMI tertile	-0.05	1.07	7.3	88.3	4.3
	Prepregnancy BMI	Middle BMI tertile	-0.01	1.06	6.6	88.1	5.3
	Prepregnancy BMI	Upper BMI tertile	0.06	0.86	1.5	94.9	3.5
	Center	Mangiagalli	-0.06	0.98	4.9	91.2	3.9
	Center	Monza	0.23	1.03	3.7	89.9	6.4
	Center	Trieste	-0.26	0.92	11.8	86.0	2.1
Lean mass							
	Gestational age	14-27 wk	0.01	1.00	4.5	90.2	5.2
	Gestational age	8-13 wk	0.01	1.00	5.0	89.2	5.8
	Gestational age	>=28 wk	-0.01	1.00	5.7	88.4	5.9
	Prepregnancy BMI	Lower BMI tertile	-0.05	0.92	5.3	91.2	3.5
	Prepregnancy BMI	Middle BMI tertile	0.06	1.08	5.8	86.5	7.6
	Prepregnancy BMI	Upper BMI tertile	0.00	0.99	3.8	90.6	5.6
	Center	Mangiagalli	0.06	0.96	4.3	90.6	5.2
	Center	Monza	-0.28	1.03	8.2	87.4	4.4
	Center	Trieste	0.36	1.06	1.1	86.0	12.9
Total body water							
	Gestational age	14-27 wk	0.00	1.00	4.7	90.3	5.0
	Gestational age	8-13 wk	0.03	0.98	4.7	89.5	5.8
	Gestational age	>=28 wk	-0.01	1.02	5.4	89.0	5.7
	Prepregnancy BMI	Lower BMI tertile	-0.06	0.96	5.3	90.9	3.8
	Prepregnancy BMI	Middle BMI tertile	0.07	1.05	5.6	87.0	7.4
	Prepregnancy BMI	Upper BMI tertile	-0.01	0.99	3.8	91.1	5.1
	Center	Mangiagalli	0.04	1.00	5.7	89.3	5.0
	Center	Monza	-0.18	0.97	4.4	90.8	4.8

Endpoint	Level	Stratum	z mean	z SD	<5th, %	5th-95th, %	>95th, %
	Center	Trieste	0.31	0.98	0.0	89.1	10.9
Visceral fat level							
	Gestational age	14-27 wk	-0.01	0.98	4.3	92.1	3.6
	Gestational age	8-13 wk	0.04	1.03	4.2	89.2	6.6
	Gestational age	>=28 wk	-0.01	1.01	5.7	90.0	4.3
	Prepregnancy BMI	Lower BMI tertile	-0.04	1.06	5.0	88.4	6.6
	Prepregnancy BMI	Middle BMI tertile	-0.07	1.05	6.8	89.4	3.8
	Prepregnancy BMI	Upper BMI tertile	0.11	0.88	2.3	94.6	3.1
	Center	Mangiagalli	-0.01	0.99	4.5	91.1	4.4
	Center	Monza	0.16	1.02	3.4	90.5	6.1
	Center	Trieste	-0.47	0.85	10.9	89.1	0.0

Expected proportions are 5% below the 5th centile, 90% between the 5th and 95th centiles, and 5% above the 95th centile. Calibration was evaluated overall and across gestational-age strata, pre-pregnancy BMI tertiles, and centers.

Table S5. Sensitivity analyses.

Analysis	Endpoint	<5th, %	5th-95th, %	>95th, %	z mean	z SD	Max center P50 range
Broader sensitivity							
Broader sensitivity	Total body water	5.3	89.3	5.4	0.00	1.00	
Broader sensitivity	Lean mass	4.5	89.7	5.7	0.00	1.00	
Broader sensitivity	Fat mass	5.2	90.1	4.6	-0.00	1.00	
Broader sensitivity	Visceral fat level	4.8	90.7	4.5	0.00	1.00	
Center-adjusted sensitivity							
Center-adjusted sensitivity	Total body water	5.5	89.4	5.2	0.00	1.00	1.8
Center-adjusted sensitivity	Lean mass	5.2	89.6	5.2	0.00	1.00	3.0
Center-adjusted sensitivity	Fat mass	5.1	90.2	4.7	-0.00	1.00	1.6
Center-adjusted sensitivity	Visceral fat level	4.4	90.7	4.9	0.00	1.00	1.3

The broader sensitivity analysis retained pregnancies with gestational diabetes or other recorded obstetric complications among pregnancies without hypertensive disorders, fetal growth restriction, or chronic hypertension. The center-adjusted sensitivity analysis refitted the primary models with center included as a fixed-effect covariate in the location parameter.

Table S6. Longitudinal models and covariate effects.

Location covariate	Estimate	95% CI	P value
Total body water (L)			
Pre-pregnancy BMI, per 1-unit increase	0.43	0.35 to 0.52	<.001
Pre-pregnancy smoking: yes vs no	0.17	-0.58 to 0.92	.653
Lean mass (kg)			
Pre-pregnancy BMI, per 1-unit increase	0.56	0.40 to 0.71	<.001
Pre-pregnancy smoking: yes vs no	0.14	-1.28 to 1.55	.850
Fat mass (kg)			
Pre-pregnancy BMI, per 1-unit increase	1.93	1.82 to 2.03	<.001
Pre-pregnancy smoking: yes vs no	0.30	-0.41 to 1.00	.407
Visceral fat level (a.u.)			
Pre-pregnancy BMI, per 1-unit increase	0.95	0.88 to 1.02	<.001
Pre-pregnancy smoking: yes vs no	0.17	-0.22 to 0.57	.393

Estimates are from the location parameter of longitudinal models fitted in the 442-patient reference population and are expressed in the units indicated for each endpoint. Box-Cox t marginal distributions with constant scale and shape parameters were linked through a Gaussian copula with an unstructured correlation matrix across the three gestational periods. The location parameter included a gestational-age spline, linear pre-pregnancy BMI, and pre-pregnancy smoking status. The cohort, covariates, and spline basis were identical to those used for the primary marginal-model comparison.
